# Integrating research infrastructures into infectious diseases surveillance operations: Focus on biobanks

**DOI:** 10.1016/j.bsheal.2022.10.001

**Published:** 2022-12

**Authors:** Plebeian B. Medina, Jennifer Kealy, Zisis Kozlakidis

**Affiliations:** a Research Institute for Tropical Medicine – Department of Health, Manila, Philippines; b London School of Hygiene and Tropical Medicine, London, UK; c International Agency for Research on Cancer/World Health Organization, 150 Cours Albert Thomas, 69372 Lyon CEDEX 08, France

**Keywords:** Biobanking, Research infrastructures, Infectious diseases, Surveillance, Integration

## Abstract

Technological advances in the first two decades of the 21^st^ century have profoundly impacted medical research in many ways, with large population cohorts, biological sample collections and datasets through biobanks becoming valued global resources to guide biomedical research, drug development, and medical practice. However, in order for biobanks to maximize their impact and scientific reach of their resources, they would need to act within a complex network of infrastructures and activities. Therefore, different ways have emerged in which biobanks, including those for infectious diseases, can emerge as (part of) infrastructures, integrate within existing ones, or become an independent, yet an interoperable component of the existing infrastructural landscape. However, there has been a limited understanding and study of such mechanisms to date. This perspective aims to address this knowledge gap and illustrates these three high-level ways in which such infrastructures could integrate their activities and identifies the necessary key pre-conditions for doing so, while drawing from specific examples.

## 1 Introduction

Technological advances in the first two decades of the 21st century have profoundly impacted medical research in many ways. Following the completion of the Human Genome Project, genomic research progressed rapidly, with large datasets and reference maps becoming valued global resources to guide scientific and clinical research, drug development, and medical practice [1]. This push for the availability of scientific reference data sets, often within the framework of open data science, has subsequently led to the creation of a variety of dedicated research infrastructures that gather data and provide services to different research communities. The European Research Infrastructures are one such example, involving research areas such as paediatrics [Paediatric Translational Research Infrastructure (ID-EPTRI); https://eptri.eu/], biobanking [Biobanking and Bio-Molecular resources Research Infrastructure (BBMRI); https://www.bbmri-eric.eu/] and others.

In the case of biobanking, large-scale population biobanks, such as the UK Biobank, should be best viewed as research platforms rather than discrete research projects [2]. They comprise large volumes of research participants, may be designed to run for long periods of time (possibly decades), and encompass numerous separate research projects over their lifespan, for example the European Prospective Investigation into Cancer and Nutrition (EPIC; https://epic.iarc.fr/) at the International Agency for Research on Cancer (IARC), as well as the European Virus Archive (EVAg; https://www.european-virus-archive.com/). As such, biobanks are considered foundational infrastructures for medical research [3]. However, in order for biobanks to maximize their impact and scientific reach of their resources, they need to foster their interoperability, both with other biobanks, as well as other existing research infrastructures [4].

**Fig. 1:**
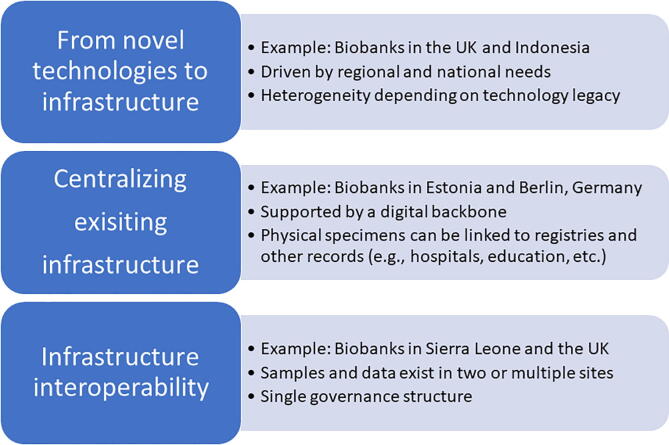
Three approaches for integrating research infrastructure activities, including biobanking.

As examples of such infrastructures in the field of infectious diseases, in 2015 the World Health Organization (WHO) unveiled a Global Action Plan on anti-microbial resistance (AMR), supported by the Global Antimicrobial AMR Surveillance System (GLASS; https://www.who.int/initiatives/glass). The aims of the WHO AMR surveillance programme include monitoring trends in infection and resistance to develop standard treatment guidelines. This AMR surveillance should provide early alerts for the emergence of novel resistant strains and aid the rapid identification and control of outbreaks. Almost in parallel, a similar mechanism was unveiled for Influenza, under the Global Initiative on Sharing All Influenza Data (GISAID; https://www.gisaid.org/), which more recently was utilised as research infrastructure for SARS-CoV-2 data as well [5]. These two infrastructures do not contain any physical samples, and neither can link to physical samples in any biobanks. This perspective illustrates three high-level ways in which such infrastructures could integrate their activities and identifies the necessary key pre-conditions for doing so, while drawing from specific examples, as illustrated in Fig. 1.

## 2 Approaches for integrating research infrastructure activities

Infrastructures have always stood as a means to an end: being built, maintained, and expanded in order to enable the functioning of specific sets of activities. As they refer to sets of activities, they are comprised of a number of technical building blocks reflecting professional, regional, and societal needs. At the same time, as infrastructures represent long-term commitments toward achieving a particular aim, this also means that certain resources and uses will be locked-in for the longer-term [6].

2.1. ***Joining novel technologies into emerging infrastructures (bottom-up approach)***:
  The use of antiquated infrastructures for the collection of and access to biological samples and medical data, can lead to financial and operational inefficiencies, and sources of error [7]. Therefore, the creation of better-performing, sustainable infrastructures improving or replacing existing ones is often sought [8]. One of the ways in which such new infrastructures can emerge is by bringing together technologies under a single umbrella (e.g., digital records, genomics, automation, and biobanking). In healthcare, one example is the considerable investment in the field of electronic health records, where computerized information and communication technologies have been introduced. In the United Kingdom (UK) for example, there has been a consistent effort over the last two decades to drive efficiencies into the national universal healthcare system [9] using digital approaches. However, the non-standardized healthcare systems in the UK, developed through regional procurement, needs, and decisions, meant that the relative success of integrating such new technologies into existing infrastructures was context-driven as much as needs-driven – and not necessarily transferable across all geographies [10]. Thus, biobanks are integrated into specific local healthcare systems (with their individual IT/LIMS systems), rather than a single comprehensive national infrastructure [11], while the UK Biobank has interoperability solutions with those infrastructures, but is not integrated into them [12]. In contrast, the deployment of a digital national surveillance program for COVID-19 in Indonesia was more successful, as it did not have to accommodate legacy systems when new technologies were introduced as part of a single surveillance infrastructure [13]. This success paves the way for expanding the implementation to the surveillance of other infectious diseases as part of ongoing national programs, and to linking with the hospital-based biobanks -that represent the majority of Indonesian biobanks- within a harmonized framework [14].
2.2. ***Centralizing existing infrastructures (top-down approach)*:**
  A different approach to create infrastructures is to push for consolidated biomedical and research laboratories, where resources and services are centralized and serve a large(r) population for the purposes of enhanced efficiency, increased standardization, and potentially earlier time to results. This approach is often driven by business considerations and scarcities of appropriately qualified personnel, while additional secondary benefits, can include integrated databases, reporting systems as well as biobanks [15]. In contrast to the UK example above, Estonia has taken a different approach and centralized several different infrastructures into a single one. Estonia’s digital national infrastructure, where state services are digital, includes virtually all interactions a citizen can have with the government (e.g., vehicle registration, population registration, etc.) The benefit of such an approach is not only that every citizen has digital access to government and certain private-sector services, but also that all of these services communicate with each other and can exchange data to accomplish complex tasks. For example, a person’s health record is queried for suitability when that person renews their driver’s license [16]. The biobanking infrastructure in Estonia is also part of this digital environment, where physical specimens in the biobank are linked to the health registries which can talk to one another [17], [18]. This enables researchers to find well-characterized specimens. Regarding diagnostic services linked to biobanking, Labor Berlin Services serves as a characteristic example. It was created in 2011 by the centralization of the two main healthcare networks in the state of Berlin, Germany. The new centralized infrastructure performs over 8 million bacteriological analyses per year. Additionally, as the diagnostic laboratory of the largest University Hospital in Europe (Charité), it could build a strong research and development platform, i.e., setting up a diagnostic clinical scientist program (2012) and linked biobank (2013) to attract promising young microbiologists, virologists, and laboratory medicine specialists [19].
2.3. ***Interoperability of existing infrastructures (cross-cutting approach)*:**
  In some cases, neither one of the above approaches is appropriate, i.e., centralization of existing infrastructure(s) or creation of new infrastructure(s), For example when there is an insufficient capital investment to support such large-scale implementation of changes, then an alternative solution is sought. It should be noted that it is not necessary or practical to always pursue the creation of a single global infrastructure – though in some contexts it is clearly easier to create, e.g., in small countries such as Estonia - to avoid incompatible data or data sharing: data standardization and interoperability of existing infrastructures would suffice. In certain cases, a unique infrastructure could even be worse than a federation of compatible, interoperable infrastructures: as the former might be difficult to manage, particular when considering other aspects such as general regulations and ground operations in regional de-centralized political structures, such as Länder in Germany or Regions in Italy. Furthermore, interoperability of existing infrastructures can be particularly useful in Low-and-Middle-Income countries, with limited resources. During the Ebola outbreak in Sierra Leone (2014–2015) residual clinical specimens and accompanying data were collected in the field, for routine diagnostic testing in Public Health England (PHE)-led laboratories. For biobanking purposes, after the epidemic ended, some of these samples remained in Sierra Leone to facilitate further research opportunities, while the majority of these samples, with all the accompanying data, were transferred to PHE laboratories in the UK (now renamed as UK Health Security Agency) for curation by PHE in dedicated facilities. Then, jointly the Ministry of Health and Sanitation and Public Health England (MOHS-PHE) Ebola Biobank created a single governance group, including representatives from different stakeholders, to streamline the utilization of this interoperable infrastructure, as samples are now accessible through this single governance structure [20]. This approach was also adopted in the case of linking existing surveillance programs with biobanking for infectious diseases of companion animals in the UK. Specifically, it linked the existing Small Animal Veterinary Surveillance Network (SAVSNET) to the diagnostic laboratories, so that identified, targeted, residual diagnostic samples of interest can be retained in a dedicated biobank for future research [21].

## 3 Considerations

It has to be noted at this point, that field activity in infectious diseases, such as the collection of specimens and/or pathogens, follows standardized procedures that aim to guarantee the compatibility of samples collected in different conditions. The same holds true for analyses, however here the digital outcome may differ due to different instruments, a different way of interpreting the results, and so on. Thus, standardization relies mainly on shared procedures, which include how to deal with the characteristics of analytical equipment and their digital outcomes: for example, data from instruments of different precision cannot be compared in a straightforward way. Finally, a compatible data organization is necessary to guarantee full interoperability among different (data) infrastructures, in what is usually called 'semantic interoperability': it is necessary that data stored in infrastructure A mean the same as data stored in infrastructure B, to enable A and B to dialogue and effectively co-operate; or, at least, a bi-lingual vocabulary - in technical terms, a 'mapping' - is required, associating the logical structures of the two data banks [22], [23].

There are a number of ways in which technologies and infrastructures in healthcare and biomedical research can be integrated. Given the multitude of active biobanks, healthcare, and research infrastructures, it is most likely that any such integration would not comprise a single, uniform approach, but rather would be variable, depending on a number of parameters. These parameters include, but are not limited to: i) The levels of *heterogeneity*, as infrastructures are complex systems, different professionals would need to come together to achieve pre-defined goals. This is already the case with biobanking providing services to wide sets of research activities-but that might be amplified within a larger infrastructure setting [24]. ii) The levels of *standardization*, especially in the technical components, as for example standardization in data capture and sharing would be critical both for integration into infrastructures, as well as any interoperability between infrastructures [4]. iii) The *regulatory landscape*, as it would need to be aligned with existing and proposed regulations for both physical specimens and data [25]; iv) The *sustainability*, as resources would be required for supporting operations and maintenance in the longer term, while transparent conduct will maintain the social aspects that have been identified as a key component in biobanking sustainability [26]; and v) The *biosecurity*, protecting the collected samples and staff from any untoward activity.

This manuscript has certain limitations, in an effort to create a high-level overview, it does not distinguish among the different components of infrastructure. Infrastructures consist of fieldwork, laboratory activities and in the management of data resulting from them, and integration or merging can take place for such constituent activities, without necessarily reflecting the organization of the entire infrastructure. Additionally, the different parameters considered above have been listed briefly, however each one merits to be addressed specifically and in-depth.

## 4 Conclusion

The future of continually evaluating and improving healthcare services relies on establishing infrastructures to respond to healthcare needs at scale, such as infectious diseases surveillance operations, research institutions, biobanks, etc., and to foster the interaction between such infrastructures and any new technologies that might arise. Therefore, infrastructure should not be viewed simplistically, comprised only of a set of discrete technologies. Instead, infrastructure should be seen as a complex, interconnected system of technology embedded in professional activities, society, and the environment, interacting with public and private institutions [27]. To this end, existing policy frameworks on technological integrations could be adapted specifically to biobanking and be beneficial in fostering the long-term development in the biobanking field. For example, developing policies for the integration of open-source technologies using standardized data formats means that as technology advances, it relies on open standards and common formats rather than proprietary formats, allowing for a greater degree of transparency as the infrastructure ages.

A future demand might arise for integrating biobanks into existing infrastructures, indirectly through biobank networks, such as BBMRI-ERIC or the International Society for Biological and Environmental Repositories (ISBER). For example, in the case of COVID-19 BBMRI and ISBER embarked on a joint initiative to facilitate access to samples and relevant data and facilitate research [28]. Being able to join such research infrastructures to infectious diseases surveillance operations might be indeed beneficial both for research and clinical needs. However, as there are additional layers of interoperability required, perhaps the complexity of doing so currently represents a formidable challenge.

As both biobanks and infectious diseases surveillance operations reach maturity, wider reach and impact, it is more than likely that novel ways of interaction between the two might become necessary. Understanding the ways in which these might be possible will support the future expansion and scaling up of activities, especially as new technologies and new fields of research and healthcare work become embedded into existing infrastructures.

## References

[1] Rood J.E., Regev A. (2021). The legacy of the Human Genome Project. Science.

[2] Bycroft C., Freeman C., Petkova D., Band G., Elliott L.T., Sharp K., Motyer A., Vukcevic D., Delaneau O., O’Connell J., Cortes A. (2018). The UK Biobank resource with deep phenotyping and genomic data. Nature.

[3] Kinkorová J. (2016). Biobanks in the era of personalized medicine: objectives, challenges, and innovation. EPMA J..

[4] Kiehntopf M., Krawczak M. (2011). Biobanking and international interoperability: samples. Human Genet..

[5] GISAID (Global Initiative on Sharing All Influenza Data), GISAID mission. https://www.gisaid.org/about-us/mission, 2020 (accessed on 25 February 2022).

[6] Zhang D., Yu Z., Chin C.Y. (2005). Context-aware infrastructure for personalized healthcare. Stud. Health Technol. Inf..

[7] Kaufman J.H., Eiron I., Deen G., Ford D.A., Smith E., Knoop S., Kol T., Mesika Y., Witting K., Julier K. (2005). From regional healthcare information organizations to a national healthcare information infrastructure. Perspect. Health Inf. Manag..

[8] Long C.M., Marzi A. (2021). Biodefence research two decades on: worth the investment?. Lancet Infect. Dis..

[9] Sheikh A., Jha A., Cresswell K., Greaves F., Bates D.W. (2014). Adoption of electronic health records in UK hospitals: lessons from the USA. Lancet.

[10] M. Willis, University of Oxford. National digital infrastructures for healthcare: A comparative case of Estonian and British healthcare infrastructure, https://www.politics.ox.ac.uk/node/4704, 2018 (accessed on 26 February 2022).

[11] McLauchlan J., Innes H., Dillon J.F., Foster G., Holtham E., McDonald S., Wilkes B., Hutchinson S.J., Irving W.L. (2017). HCV Research UK Steering Committee. Cohort profile: the hepatitis C virus (HCV) research UK clinical database and biobank. Int. J. Epidemiol..

[12] Adamska L., Allen N., Flaig R., Sudlow C., Lay M., Landray M. (2015). Challenges of linking to routine healthcare records in UK Biobank. Trials.

[13] Aisyah D.N., Mayadewi C.A., Budiharsana M., Solikha D.A., Ali P.B., Igusti G., Kozlakidis Z., Manikam L. (2022). Building on health security capacities in Indonesia: Lessons learned from the COVID-19 pandemic responses and challenges. Zoonoses Public Health.

[14] Fachiroh J. (2020).

[15] Vandenberg O., Durand G., Hallin M., Diefenbach A., Gant V., Murray P., Kozlakidis Z., van Belkum A. (2020). Consolidation of clinical microbiology laboratories and introduction of transformative technologies. Clin. Microbiol. Rev..

[16] Adeodato R., Pournouri S., (Eds.) (2020). InCyber Defence in the Age of AI, Smart Societies and Augmented Humanity.

[17] Leitsalu L., Alavere H., Tammesoo M.L., Leego E., Metspalu A. (2015). Linking a population biobank with national health registries—the Estonian experience. J. Personalized Med..

[18] Leitsalu L., Metspalu A. (2017). From biobanking to precision medicine: the estonian experience, InGenomic and precision medicine.

[19] Hummel M., Specht C. (2019). Biobanks for future medicine. J. Lab. Med..

[20] Hannigan B., Whitworth J., Carroll M., Roberts A., Bruce C., Samba T., Sahr F., Coates E. (2019). The Ministry of Health and Sanitation, Sierra Leone-Public Health England (MOHS-PHE) Ebola Biobank. Wellcome Open Res..

[21] Smith S.L., Afonso M.M., Roberts L., Noble P.J., Pinchbeck G.L., Radford A.D. (2021). A virtual biobank for companion animals: A parvovirus pilot study. Veterinary Record..

[22] Merino-Martinez R., Norlin L., van Enckevort D., Anton G., Schuffenhauer S., Silander K., Mook L., Bild R., Swertz M., Litton J.E. (2016). Toward global biobank integration by implementation of the minimum information about biobank data sharing (MIABIS 2.0 Core). Biopreservation and biobanking.

[23] Lablans M., Bartholomäus S., Ückert F. (2011). Providing trust and interoperability to federate distributed biobanks. InUser Centred Networked Health Care.

[24] Jiang C.Q., Lam T.H., Lin J.M., Liu B., Yue X.J., Cheng K.K., Tomlinson B., Wong K.S., Cheung B.M., Thomas G.N. (2010). An overview of the Guangzhou Biobank Cohort Study-Cardiovascular Disease Subcohort (GBCS-CVD): A platform for multidisciplinary collaboration. J. Human Hypertens..

[25] Rial-Sebbag E., Cambon-Thomsen A. (2012). The emergence of biobanks in the legal landscape: towards a new model of governance. J. Law Soc..

[26] Watson P.H., Nussbeck S.Y., Carter C., O’Donoghue S., Cheah S., Matzke L.A., Barnes R.O., Bartlett J., Carpenter J., Grizzle W.E., Johnston R.N. (2014). A framework for biobank sustainability. Biopreservation Biobanking.

[27] Roelich K., Knoeri C., Steinberger J.K., Varga L., Blythe P.T., Butler D., Gupta R., Harrison G.P., Martin C., Purnell P. (2015). Towards resource-efficient and service-oriented integrated infrastructure operation. Technol. Forecast. Soc. Change.

[28] Catchpoole D.R., Florindi F., Ahern C., Garcia D.L., Mullins P., Van Enckevort E., Zaayenga A., Mayrhofer M.T., Holub P. (2020). Expanding the BBMRI-ERIC Directory into a Global Catalogue of COVID-19–Ready Collections: A Joint Initiative of BBMRI-ERIC and ISBER. Biopreservation Biobanking.

